# Dynamic Contrast-Enhanced Ultrasound of Colorectal Liver Metastases as an Imaging Modality for Early Response Prediction to Chemotherapy

**DOI:** 10.3390/diagnostics7020035

**Published:** 2017-06-12

**Authors:** Marie Benzon Mogensen, Martin Lundsgaard Hansen, Birthe Merete Henriksen, Thomas Axelsen, Ben Vainer, Kell Osterlind, Michael Bachmann Nielsen

**Affiliations:** 1 Department of Oncology, Copenhagen University, Rigshospitalet, Copenhagen 2100, Denmark; Kell.Oesterlind@regionh.dk; 2 Department of Radiology, Copenhagen University, Rigshospitalet, Copenhagen 2100, Denmark; martin.lundsgaard.hansen@regionh.dk (M.L.H.); birthe.merete.henriksen@regionh.dk (B.M.H.); thomas.axelsen@regionh.dk (T.A.); Michael.Bachmann.Nielsen@regionh.dk (M.B.N.); 3 Department of Pathology, Copenhagen University, Rigshospitalet, Copenhagen 2100, Denmark; Ben.Vainer@regionh.dk

**Keywords:** dynamic contrast-enhanced ultrasound (DCE-US), colorectal cancer, early response evaluation, liver metastases

## Abstract

Our aim was to investigate whether dynamic contrast-enhanced ultrasound (DCE-US) can detect early changes in perfusion of colorectal liver metastases after initiation of chemotherapy. Newly diagnosed patients with colorectal cancer with liver metastases were enrolled in this explorative prospective study. Patients were treated with capecitabine or 5-fluorouracil-based chemotherapy with or without bevacizumab. DCE-US was performed before therapy (baseline) and again 10 days after initiation of treatment. Change in contrast-enhancement in one liver metastasis (indicator lesion) was measured. Treatment response was evaluated with a computed tomography (CT) scan after three cycles of treatment and the initially observed DCE-US change of the indicator lesion was related to the observed CT response. Eighteen patients were included. Six did not complete three series of chemotherapy and the evaluation CT scan, leaving twelve patients for analysis. Early changes in perfusion parameters using DCE-US did not correlate well with subsequent CT changes. A subgroup analysis of eight patients receiving bevacizumab, however, demonstrated a statistically significant correlation (*p* = 0.045) between early changes in perfusion measures of peak enhancement at DCE-US and tumor shrinkage at CT scan. The study indicates that early changes in DCE-US perfusion measures may predict subsequent treatment response of colorectal liver metastases in patients receiving bevacizumab.

## 1. Introduction

Colorectal cancer (CRC) is the third most common type of malignancy and the fourth most common cause of death due to cancer [1]. During the last decade, major progress in the treatment of disseminated CRC has occurred, especially with systemic treatment supplemented with surgical resection of liver and lung metastases for selected subgroups. Thus, distant metastases are not necessarily an obstacle of cure, provided the metastases are resectable or can become resectable by neoadjuvant treatment [1]. It is therefore of utmost importance to optimize both treatment and evaluation methods. Angiogenesis is mandatory for a tumor to grow, which is why drugs neutralizing vascular endothelial growth factor (VEGF), e.g., bevacizumab, have become part of the treatment of metastasizing CRC (mCRC).

The standard method for evaluation of treatment response is a computed tomography (CT) scan looking for tumor shrinkage as defined in the Response Evaluation Criteria In Solid Tumors (RECIST), version 1.1 [2]. Since the RECIST criteria are based solely on tumor size, they do not account for structural changes as reflected by radiological enhancement or attenuation, and the RECIST criteria do not discriminate necrosis or scarring from vital tumor tissue. Targeted drugs do not necessarily result in immediate tumor shrinkage, and the regular RECIST evaluation is therefore not always sufficient [3]. Functional imaging has consequently received increasing attention, opening possibilities of detecting early signs of response prior to macroscopic shrinkage. This may make it possible to spare patients with unresponsive tumors of futile treatment and shift to another regime, in an aim to improve the overall treatment outcome.

Dynamic, contrast-enhanced ultrasound (DCE-US) is a functional imaging modality with a potential for tumor response assessment. The method involves continuous ultrasound scanning during contrast circulation, resulting in time-intensity curves for contrast enhancement. The curves can be analyzed by mathematical perfusion models [4]. The procedure can be repeated without exposing the patient to radiation and is not nephrotoxic, which for some cancer patients can be essential [5]. Compared to dynamic magnetic resonance imaging and dynamic CT, ultrasound has the advantages of high temporal and spatial resolution [6]. Ultrasound contrast agents are strictly intravascular and therefore reflect perfusion in the vascular bed of the selected tissue [7]. Prior studies have shown promising results with this technique in regards to response assessment [8,9,10].

The aim of this study was to investigate whether early changes in perfusion measured by DCE-US predicts the response to treatment of colorectal liver metastases, and whether the modality can be applied as a tool for individualizing treatment.

## 2. Materials and Methods

### 2.1. Patient Population

Patients referred to Department of Oncology at Rigshospitalet, Copenhagen University Hospital, for treatment of newly diagnosed metastatic CRC in the period June 2012 until January 2014 were considered for inclusion. Inclusion criteria were at least one measurable liver metastasis of more than one centimeter, a histologically confirmed primary colorectal adenocarcinoma, no previous cancer diagnosis, and a performance status of 0–1 (i.e., their general condition should allow for work of a light or sedentary nature) [11]. Furthermore, the patients should be candidates for and accept first-line chemotherapy according to the local guidelines. Patients without mutation in the *RAS* gene were treated with 5-fluorouracil (day 1–3 + 15–17) and irinotecan (day 1 + 15) (FOLFIRI) plus cetuximab (day 1 + 15), whereas patients with a *RAS* mutation were treated with capecitabine (day 1–14) and oxaliplatin (day 1) (CAPOX) with bevacizumab (day 1) added, as long as contraindications such as surgery within the previous month or not well-regulated hypertension were absent. Additional exclusion criteria were acute myocardial infarction or unstable angina pectoris within the last six months. Each treatment cycle consisted of 21 days in the treatment regimen of CAPOX while FOLFIRI lasted 28 days. Only patients receiving at least three series of chemotherapy followed by a CT scan were eligible for correlation analysis between early DCE-US measures and subsequent conventional CT response evaluation. This explorative prospective study was conducted according to the Helsinki declaration and the study protocol was approved by the Regional Ethics Committee of The Capital Region of Denmark (H-3-2012-041, 1 June 2012). All the patients provided written consent.

### 2.2. Imaging Protocol

All ultrasound (US) examinations were performed using a GE Logiq e9 navigation system (GE Medical Systems, Milwaukee, WI, USA). The contrast agent used was SonoVue (Bracco, Italy). First, a morphologic examination was performed in B mode to select an appropriate tumor target. Investigations were only performed on one metastasis even in livers where several were present. The choice of a metastasis for DCE-US measurements was based on the criteria that it should be visible and completely contained and surrounded by healthy appearing liver tissue in the ultrasound image in a sagittal scan plane below the rib curvature during the respiratory cycle. If several were present the largest was chosen. Each patient was instructed to fast for at least two hours prior to the US scanning to standardize portal venous flow. All US examinations were performed by the same radiologists (MLH and BMH (+20 years of experience)). Intravenous contrast was given manually as a bolus of 1.4 mL plus a saline flush of 10 mL over 5 s. The patients were instructed to breathe shallowly during a 2 min recording of a cineloop sequence over the selected metastases. The CINE sequences were saved in DICOM file format for subsequent analyses.

DCE-US was performed as baseline before initiation of treatment (median 4 days), and early evaluation DCE-US were made on Day 10 (with the first day of treatment referred to as Day 1) (median 10, range 9–13). Baseline CT was performed no earlier than a week before treatment initiation (median 5 days before, range 2–6). After three cycles of treatment, a conventional CT scan with intravenous contrast injection was performed for response evaluation. Evaluation at Day 10 was chosen because a treatment-induced reduction in tumor perfusion is supposed to peak early, which has been shown at Day 7 in a study on GIST tumors treated with Imatinib [10].

### 2.3. Imaging Analysis

**Figure 1:**
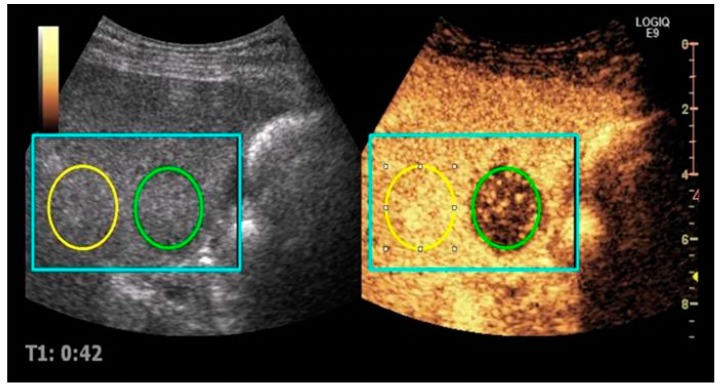
Split view of simultaneous imaging with contrast enhanced imaging on the right and B-mode on the left. Region of interest (ROI) is placed within the metastasis (green) and in normal liver tissue (yellow). The blue frame illustrates analysis area with motion correction.

All US images were analyzed by one radiologist (MLH) blinded from treatment information and using commercially available software (Vuebox version 3; Bracco, Italy). A specific calibration file provided by the vendor for GE Logiq e9 was used in the analysis software to convert ultrasound images to linearized data for time-intensity curve analysis. A circular region of interest (ROI) was drawn within the demarcation margins of the selected liver metastasis as illustrated in Figure 1. Thus, the ROI only contained metastatic tissue, and its size varied with that of the metastasis. It was automatically positioned by the software to adjust for respiratory motion on the following images of the sequence. Furthermore, a similar sized ROI was positioned in normal liver tissue without visible large vessels at the same depth as a reference for normalization of the measurement. Time-intensity curves were generated both for metastatic and for normal tissue.

**Figure 2:**
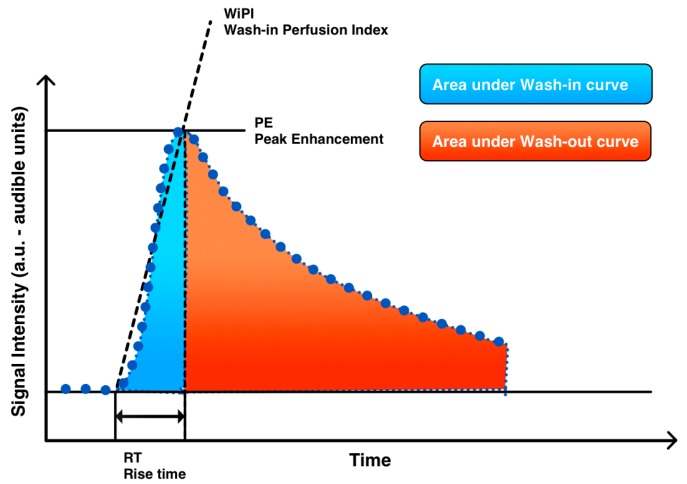
Fitted time-intensity curve illustrating measured parameters of peak enhancement (PE), area under the wash-in curve, area under the wash-out curve (WiAUC), wash-in perfusion index (WiPI) and rise time (RT). WiPI is WiAUC divided by RT being the interval from the beginning until peak enhancement.

Values of the following parameters were derived from the time-intensity curves (Figure 2): peak enhancement (PE), rise time (RT), area under the curve of wash-in (WiAUC), wash-in perfusion index (WiPI) and area under the complete wash-in/wash-out curve (WiWo). PE was defined as the maximum intensity of the signal produced by the contrast agent. WiAUC was defined as the area under the curve from start until peak enhancement. WiPI was defined as the area under the curve during wash-in divided by the rise time, i.e., the interval from beginning enhancement until peak enhancement (WiPI = WiAUC/RT). WiWo was defined as the area under the curve from the start of the enhancement to end of the recorded CINE sequence. Perfusion measures in the metastases were standardized (*n*) to measures in normal liver tissue by the ratio

nPE = PE (Metastases)/PE (Normal tissue)

and correspondingly: nWiWo, nRT, nWiR, nWiPI, nWiAUC.

The liver metastases examined with DCE-US were in accordance with the RECIST criteria measured for response by taking the longest diameter on the CT scan performed after three cycles of treatment by a radiologist (TA) blinded for measures from DCE-US. Shrinkage was expressed as the percentage of change of the longest diameter (LD). Furthermore, DCE-US measures of the selected liver metastases were correlated to the CT response of the same metastasis.

### 2.4. Statistical Analysis

Based on a confidence level of 95%, a power of 80% and an expected correlation coefficient of 0.6 a sample size of 19 patients was needed. Measures were normalized according to reference values in the surrounding, normal liver tissue to permit comparison of measures between two examinations. Wilcoxon’s signed rank sum test for paired data was used to compare changes in DCE-US parameters from baseline to early evaluation. The endpoint was the change of the longest diameter of the target tumor after three cycles. Spearman’s correlation coefficient was used to examine correlations between DCE-US perfusion parameters and CT scan changes in the entire group. Due to the binary outcome of “response” versus “non-response”, logistic regression was used for correlation of changes in nPE and response. *p*-Values less than 0.05 were considered statistically significant. The positive predictive value for using early changes in nPE as predictor of later tumor growth was calculated. The cut off value was no change (0 cm) in regard to tumor growth/shrinkage as well as to changes in nPE considering an increase in nPE as predictor for growth. All statistical analyses were performed by the use of SPSS software (version 19, IBM, Armonk, NY, USA).

## 3. Results

Eighteen patients were included, but only 17 had a baseline DCE-US, as the liver metastases in one patient could not be visualized with US B-mode. Four patients did not have a second DCE-US because of disease or treatment complications, and the DCE scan of one patient was not suitable for analysis due to motion out of scan-plane. Thus, in total 12 patients were available for definitive analysis. All but one patient were treated with the CAPOX regime, of whom eight also received bevacizumab. One patient received FOLFIRI plus cetuximab. Patient characteristics are listed in Table 1.

**Figure 3:**
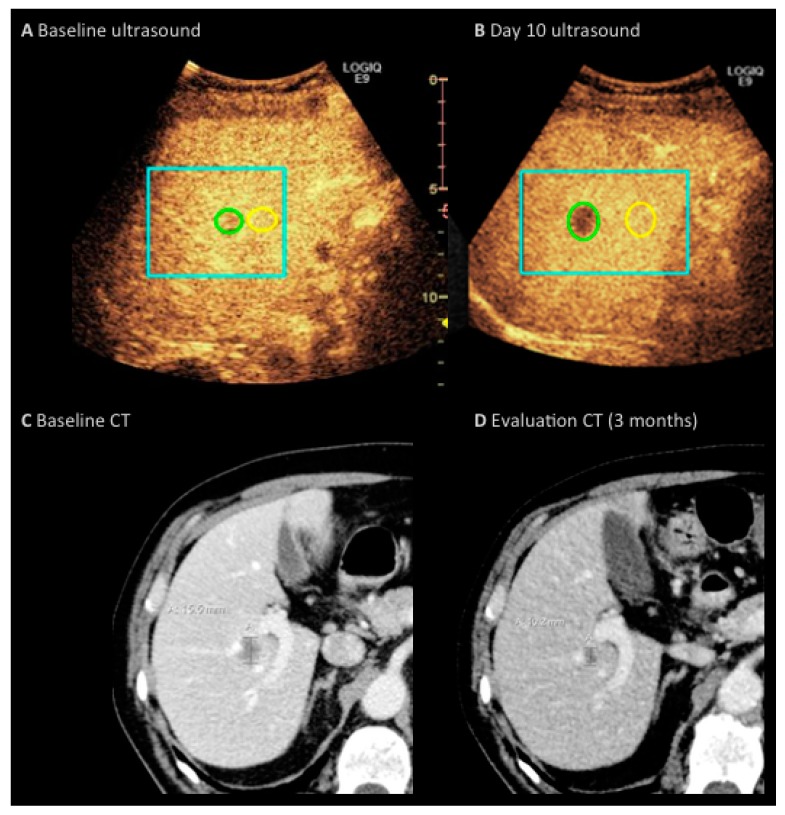
71-year-old man with metastatic colorectal cancer treated with bevacizumab, capecitabine and oxaliplatin. (**A**) baseline ultrasound scan and (**B**) Day 10 ultrasound scan. The blue frame delineates the motion corrected area; (**C**,**D**) illustrates Baseline CT and Evaluation CT with a reduction in the size of the metastasis from 16 to 10 mm on longest diameter.

The median size of ROI was 0.8 cm^2^ (range 0.2–1.7 cm^2^). Differences between metastatic and normal liver tissue were statistically significant for all perfusion parameters. CT response after three cycles of treatment resulted in one patient with progressive disease, four patients with stable disease and seven with a partial response regarding the measured metastases. None had a complete response. Spearman’s test of correlation between changes in perfusion parameters and changes in the longest diameter of the measured liver metastases showed no significant correlations: The correlation coefficient ρ was as follows: nPE (ρ = 0.455), nWiWo (ρ = 0.291), nRT (ρ = 0.223), nWiR (ρ = 0.165), nWiPI (ρ = 0.140) or nWiAUC (ρ = 0.075) (Table 2). Figure 3 shows baseline scan and Day 10 scan for a responding patient compared with the baseline and evaluation CT.

Since treatment with the anti-angiogenic drug bevacizumab is known to result in changes in the perfusion of the tumor, a subgroup analysis of the eight patients who received bevacizumab were made, showing a significant correlation between the changes in nPE and the changes on CT scan of the selected liver metastases (ρ = 0.719; *p* = 0.045). No correlation was seen when the CT changes were correlated to nWiWo (ρ = 0.108), nRT (ρ = −0.108), nWiR (ρ = 0.048), nWiPI (ρ = 0.313) or WIAUC (ρ = 0.319) (Table 2). Considering the ability of using an early increase in nPE as a predictor of tumor growth, the positive predictive value in the group of patients treated with bevacizumab was 25% while the negative predictive value was 100%.

## 4. Discussion

In this explorative study, we investigated the technical feasibility and potential clinical value of DCE-US as an early indicator of treatment effect of chemotherapy for colorectal liver metastases. Even though the number of patients in the study was limited, we found that early changes in the perfusion characteristic PE of investigated liver metastases correlated to shrinkage as evaluated on the subsequent CT scans, but only in the subgroup of patients receiving bevacizumab. This means that patients with a decrease in nPE had a high probability of response, while those showing an increase did not respond. It may be argued that a decrease in PE merely reflects the well-known pharmacologic effect of bevacizumab blocking the angiogenic signal, by which the angiogenesis is reduced. However, the correlation to the radiological tumor shrinkage much later is noteworthy.

Patients with colorectal liver metastases have the potential to be cured if the metastases can be resected at time of diagnosis or after neoadjuvant chemotherapy. Especially in situations where neoadjuvant treatment is indicated, it is of utmost importance to discontinue an ineffective treatment as early as possible and shift to another chemo-regime or go directly to surgery. The need to optimize the treatment response as well as practical limitations of evaluating the contributing effect of new targeted drugs has led to an increased interest in and efforts to develop functional imaging techniques, of which the DCE-US might have potential for evaluation of target lesions in the liver.

DCE-US is used in daily clinical practice to characterize focal liver lesions [12,13]. By qualitative analysis, real time imaging of contrast enhancement during wash-in and wash-out is used to differentiate between benign and malignant lesions [14]. For detecting colorectal liver-metastases, DCE-US has shown a diagnostic sensitivity comparable to that of CT but inferior to that of MRI [15]. In 2012, the European Federation of Societies for Ultrasound in Medicine and Biology, EFSUMB, published guidelines for performing DCE-US [7], recommending the use of DCE-US as a supplementary modality in the diagnostic setting but not as a substitute for CT or MRI. For response evaluation, Leen et al. has published a consensus paper in 2012 on DCE-US [16].

DCE-US has previously been evaluated in patients with hepatocellular carcinoma, showing that patients treated with an angiogenic inhibitor as a single-drug treatment had a decrease in the perfusion parameters after one month of treatment [17], while another study showed a change in perfusion already three days after treatment start and tended to correlate with the progression-free survival and overall survival [18]. A larger multicenter study of 539 patients with various malignancies (mostly metastatic renal cell carcinomas and only a minor fraction of disseminated CRC), all receiving antiangiogenic treatment [9], showed a correlation between the changes in perfusion parameter of area under the curve (AUC) from baseline to day 30 with freedom from progression. Focusing only on CRC with liver metastases, Schirin-Sokhan et al. [19] reported that DCE-US with the perfusion parameter of time to peak (TTP, equals RT in the present study) could predict which patients would respond to FOLFIRI plus bevacizumab based on the baseline values. Previous studies only focused on patients treated with an anti-angiogenic drug when performing early evaluation with DCE-US [20,21,22]. Neovascularization is necessary for tumor growth, so it seems understandable that changes in blood flow can reflect changes in the vitality of the tumor. DCE-US therefore seems relevant as a tool in monitoring biologically important changes in cancer treatment.

The EFSUMB 2012 paper also included recommendations for obtaining and quantifying sequential DCE-US examinations [23]. The most commonly used technique is a single bolus injection of contrast agent with continuous data acquisition over 1 to 3 min and subsequent analysis using either software on the scanner or vendor-independent stand-alone software [7]. In a large multicenter study including 2062 DCE-US examinations at 17 centers using a strict scanning protocol [23], only 3% of the examinations were considered not interpretable, which illustrates the possible clinical applicability of this modality. In order to standardize the assessments, meticulous records were kept of probe placement on skin, depth, and focus point, in order to minimize variations between scans, and bolus injections were standardized to be injected over 5 s. Recordings of the dynamic images were done during shallow breathing and preferable in a sagittal plane, so that the metastasis remained in the scanning plane during respiratory movement. Movement in the scanning plane during data acquisition allowed for subsequent motion correction supported by the software for more accurate time-intensity curves [12].

Perfusion values were normalized to healthy appearing liver tissue at the same depth as the metastases. This approach is recommended by Leen et al. [16] and been applied in similar studies [13,14]. A future practice could be similar to dynamic contrast-enhanced CT, where contrast-enhancement is normalized to an arterial input to correct for hemodynamic variations over time [24]. The size of the ROI was optimized to include as much of the metastasis as possible without the risk of including normal liver tissue at the edge of the ROI due to breathing motion. Technical problems with movement in and out of the plane during acquisition might be minimized with 3D DCE-US [25] to cover the entire lesion in all planes. This enables post-processing and the selection of relevant slices of metastatic tissue for data analysis [26]. Dynamic ultrasound images can also be difficult to obtain for metastasis in the deep part of the liver or near the diaphragm. Finally, a continuous contrast-administration using a bolus-replenish kinetic model would allow for analysis of multiple lesions in the liver within the same ultrasound examination instead of focusing on a single target lesion [23]. DCE-MRI allows multi lesion analysis, and has shown promising results in regards of monitoring the effect of bevacizumab [27]. In this study, all lesions had the same response as the one included for analysis in all patients but one. A future study might therefore benefit from including more than one lesion.

In this study, a correlation between tumor shrinkage and early changes in the perfusion parameter nPE was restricted to those receiving bevacizumab. The results should be cautiously interpreted due to the small sample size but opens for the hypothesis that early DCE-US evaluation may be useful in neoadjuvant treatment where tumor shrinkage is needed to make the tumor resectable. Four of the 12 patients had liver resections, but this was too few to relate DCE-US measures to subsequent chances of a successful resection. This observation needs validation in a larger series and the US methodology should be refined by establishing stricter scanning protocols in order to reduce data acquisition variations.

## Figures and Tables

**Table 1:** Patient characteristics.

Variable	*n* = 12	Percentage
Gender		
Female	4	33%
Male	8	67%
Age (years)	Median 66.5 (range 42–77)	
Primary tumor location		
Rectum	4	33%
Left colon	5	42%
Right colon	3	25%
Tumor burden		
Primary tumor resected	5	42%
Primary tumor in situ	7	58%
Liver metastases ≥ 3	11	92%
Liver metastases < 3	1	8%
Lymph node metastases	7	58%
Lung metastases	4	33%
Peritoneal carcinosis	2	16%
Treatment		
CAPOX + bevacizumab	8	67%
CAPOX	3	25%
FOLFIRI + cetuximab	1	8%
Carcinoembryonic Antigen Baseline	Median 52 (range 5–3050)	
Carcinoembryonic Antigen Evaluation	Median 13 (range 5–899)	

Abbreviations: CAPOX: capecitabine and oxaliplatin; FOLFIRI: 5-fluorouracil and irinotecan.

**Table 2:** Perfusion parameters and correlations co-efficient (ρ) for each parameter and correlation to tumor shrinkage.

Perfusion Parameters	Baseline (Median)	Day 10 (Median)	ρ between Shrinkage and DCE-US Measures	Bevacizumab Subgroup: ρ between Shrinkage and DCE-US Measures
nPE	0.50 (0.15–1.24)	0.27 (0.13–1.26)	0.455	0.719 (*p* = 0.045)
nWiWo	0.21 (0.01–1.53)	5.46 (0.12–31.70)	0.291	0.108 (*p* = 0.799)
nRT	0.50 (0.30–1.22)	0.58 (0.25–1.50)	0.223	−0.108 (*p* = 0.799)
nWiPI	0.48 (0.06–1.14)	0.27 (0.11–1.70)	0.140	0.313 (*p* = 0.450)
nWiAUC	0.25 (0.03–0.71)	0.17 (0.06–0.86)	0.075	0.319 (*p* = 0.441)

Abbreviations: ρ: correlation-coefficient; nPE: normalized peak enhancement; nWiWo: normalized wash-in wash-out; nRT: normalized rise time; nWiPI: normalized wash-in perfusion index; nWiAUC: normalized wash-in area under the curve, DCE-US: dynamic contrast enhanced ultrasound.
